# Soldering Aluminum

**Published:** 1897-12

**Authors:** W. S. Bates

**Affiliations:** Chicago, Ills.


### Soldering Aluminum.

BY W. S. BATES, CHICAGO, ILLS.

Mr. President, Ladies and Gentlemen: Our subject to-day is "Soldering Aluminum." The discovery of aluminum is usually attributed to Professor Frederick Wohler, he having, in 1827, produced it in a condition which enabled him to determine many of its properties. H. St. Claire Deville, however, was the first to produce it in a state of substantial purity, which he did in 1854. In his book on aluminum, published in 1859, Deville remarks on the difficulty of soldering aluminum, and after describing many of these difficulties, he says: "But a very peculiar difficulty arises here—we know no flux to clean the aluminum." This, it seems to me, is the keynote of the whole subject, and it is this flux which I have discovered.

Aluminum is a peculiar metal. It lies between the base and noble metals, partaking of the nature of both. It has a beautiful silvery color, takes a fine polish and is permanent in the air. It is soft, malleable and ductile. It withstands the fluids of the mouth and is tarnished by the fluids of the skin (the perspiration). It withstands ordinary acids, except hydrochloric, but not alkalies. Its resistance to vegetable acids, together with its high specific heat, makes it one of the finest of metals for culinary vessels. The melting point of aluminum is between that of zinc and silver. Commercial aluminum melts at about 1150° F.

The attempts to solder aluminum have been mostly, if not entirely, in the direction of discovering an alloy which would do the work. For this purpose many alloys have been suggested; most of these contain large percentages of zinc or tin; for example, Mourey's solders containing from 80 to 94 percent of zinc, and the balance aluminum; and none of them have been satisfactory, probably owing to galvanic action between the aluminum and the solder. The only one which has been at all successful, so far as I can learn, is the solder of Joseph Richards, which is composed in round numbers of 78 percent tin, 19 zinc, 2 aluminum and $\frac{1}{4}$ of one percent phosphorus. This solder requires a careful preliminary preparation and tinning of the parts to be soldered, which is fatal to it for many uses. Its composition shows that it would be useless for dental purposes.

It has also been proposed to solder a joint by immersing it in molten tin and rubbing it briskly with a steel brush. Imagine soldering a crown or bridge by such a method. Besides aluminum was soldered with tin in 1855 by Deville, and it was found that the joints were too weak and rapidly disintegrated. It has also been proposed to electro-plate the joint with copper and then flow solder on the copper, but this is obviously impracticable in most cases. The difficulties in the soldering of aluminum may be briefly summed up as follows: 1st. A thin invisible film of oxide is formed instantaneously on a clean surface, and no flux has heretofore been found which will remove this film. 2d. The position of aluminum in the galvanic scale is such, that galvanic action takes place between the aluminum and the solder and destroys the joint.

The first of these difficulties I have obviated by discovering the flux which removes the film of oxide. I regret that matters now pending in the Patent Office forbid my making the composition of this flux public at this time, but I hope to do so at some future meeting. I will simply say here that it is a flux which acts, and is used like other fluxes, for example, like borax in gold soldering.

The second difficulty I obviate by using a solder rich in aluminum. My first solder of this kind contained 70 percent aluminum and 30 percent tin. Samples of work done with this solder were shown some of you at Kalamazoo last spring, and one of your members, Dr. Rowley, is wearing an aluminum crown soldered with this solder, and which has been in for some months. This solder makes a perfect joint, and a very strong one. I have tested its strength by soldering two strips together end to end and pulling them apart. The aluminum broke and the joint held. The only objection to this solder is that it flows somewhat thick. To obviate this I substitute one-third of the tin by copper or silver, and thus secure a freer flow and at a lower temperature. Samples of work with this solder have been shown some of you at Battle Creek and Fort Wayne.

I have also used other solders. For example, I have used Dr. Carroll's plate-casting alloy composed of 90 percent aluminum and ten percent coin silver, and I have used ordinary plumbers' solder, half tin and half lead, also tin with five percent of copper, and many others.

In soldering aluminum to other metals, I have met with difficulties, owing to a peculiar property of aluminum. Aluminum forms two classes of useful alloys, one containing but a small percent of aluminum and the other containing but a small percent of the other metal. All alloys between these two extremes are with few exceptions brittle and worthless. Hence in soldering to other metals the aluminum of the solder is liable to alloy with the other metal and become super-saturated as it were, and brittle. With platinum this effect takes place at a temperature above the melting point of aluminum. Within permissible limits of temperature the solder does not alloy with platinum and does not flow readily on it, and it requires some skill to make the solder cover the pins of the teeth in plate work. With the new substitute for platinum, called "platinoid," I have no such difficulty. The solder flows on it readily. If we can get teeth with platinoid pins it will be as easy to make an aluminum plate with teeth soldered on it, as it now is to make such a plate of gold or other metal. I am now trying to get such teeth and hope to show you samples at some future meeting.

